# Molecular Evidence of High Proportion of* Plasmodium vivax* Malaria Infection in White Nile Area in Sudan

**DOI:** 10.1155/2016/2892371

**Published:** 2016-10-16

**Authors:** Makarim M. Adam Suliman, Bushra M. Hamad, Musab M. Ali Albasheer, Maytha Elhadi, Mutaz Amin Mustafa, Maha Elobied, Muzamil Mahdi Abdel Hamid

**Affiliations:** ^1^Department of Parasitology and Medical Entomology, Institute of Endemic Diseases, University of Khartoum, Khartoum, Sudan; ^2^Faculty of Medicine, University of Khartoum, Khartoum, Sudan; ^3^Faculty of Pharmacy, Al-Neelain University, Sudan

## Abstract

*Plasmodium falciparum* is a predominant malaria species that infects humans in the African continent. A recent WHO report estimated 95% and 5% of* P. falciparum* and* P. vivax* malaria cases, respectively, in Sudan. However many laboratory reports from different areas in Sudan indicated otherwise. In order to verify, we selected four hundred suspected malaria cases from Aljabalain area located in the White Nile state, central Sudan, and diagnosed them with quality insured microscopy and species-specific nested PCR. Our results indicated that the proportion of* P. vivax* infections among suspected malaria cases was high. We found that on average 20% and 36.5% of malaria infections in both study areas were caused by* P. vivax* using both microscopy and PCR, respectively. This change in pattern is likely due to the recent demographic changes and high rate of immigration from neighbouring countries in the recent years. This is the first extensive clinical study of its kind that shows rising trend in* P. vivax* malaria cases in White Nile area, Sudan.

## 1. Background

Malaria is an infectious disease of humans and other animals caused by* Plasmodium* parasites [1]. According to the latest WHO estimates, there were about 198 million cases of malaria in 2013 (with an uncertainty range of 124 million to 283 million) and an estimated 584 000 deaths (with an uncertainty range of 367 000 to 755 000) in the world [2]. Malaria mortality rates have fallen by 47% globally since 2000 and by 54% in the WHO African Region [2].

Five species of plasmodia can infect humans. The vast majority of deaths in Sub-Saharan regions are caused by* P. falciparum*, while* P. vivax*,* P. ovale*,* P. malariae,* and* P. knowlesi* cause generally milder form of malaria which other than* P. knowlesi* is rarely fatal [1, 3].

*P. vivax* is responsible for most malaria cases in Asia and Latin America but it is almost absent from most of central Africa due to the absence of Duffy antigen, the receptor which* P. vivax* uses to invade human erythrocytes [4]. In eastern and southern Africa,* P. vivax* represents around 10% to 40% of malaria cases but <1% of cases in western and central Africa [4, 5].

In Sudan until recently the majority of malaria cases were caused by* P. falciparum*.* P. vivax* is relatively rare; 95% of cases are caused by* P. falciparum* and the other 5% are caused by* P. vivax* [6]. However, in recent years many clinicians observed recurrent relapses of malaria infections in different areas in Sudan suggesting perhaps a higher than expected transmission of non-falciparum malaria parasites (most likely* P. vivax* since it is the second most important malaria parasite species in Sudan). The objective of this study was to document the suggested rise in the proportion of* P. vivax* infections among suspected malaria cases in White Nile state in Sudan.

## 2. Materials and Methods

### 2.1. Ethical Considerations

The study was approved by the ethical committee of the Institute of endemic diseases, University of Khartoum. Informed consent was obtained from each patient before participation in the study.

### 2.2. Study Area and Sample Collection

**Figure 1:**
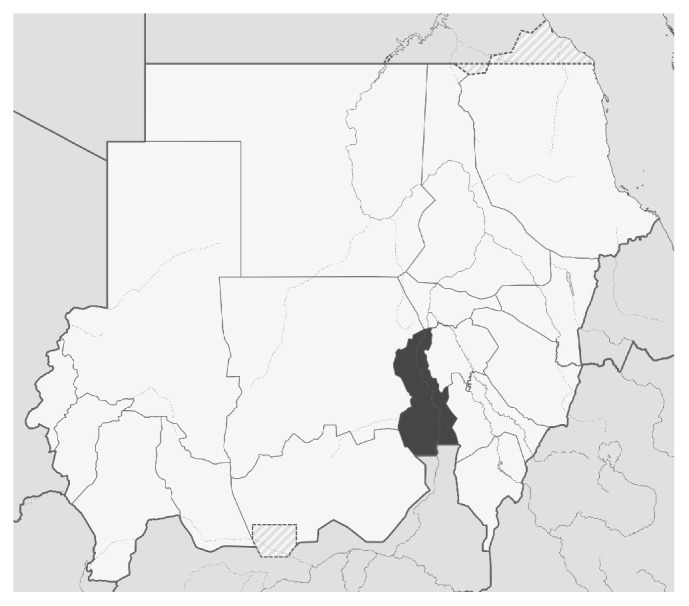
Study site: White Nile area in Sudan.

This study was a cross-sectional study carried out in the White Nile area which is one of the central Sudan states, Figure 1. It lies between latitudes 33 and 30-31 north and longitude 13 and 30–12 east, occupying an area of around 675000 km^2^, with population of 1.675 million. The study was conducted in Aljabalain area, 80 km south of Rabak town, the capital of White Nile state. The area is considered mesoendemic for malaria; transmission follows mainly the rainy season (July to October). Four hundred suspected malaria cases based on patient's symptoms (fever, headache, sweating, nausea, and vomiting) and signs (pyrexia, pallor) were chosen randomly from Aljabalain hospital and Aljabalain military hospital aged >1 year old regardless of gender. Samples were collected during the rainy season (July–September) in 2012. All suspected malaria cases were treated with a standard regimen (artemisinin based combination therapy) by the local malaria control program according to the Sudanese Malaria Treatment Guidelines in Sudan National Malaria Control Program (NMCP), and the National Protocol for Treatment of Malaria (Federal Ministry of Health, Khartoum, Sudan, 2013, unpublished).

### 2.3. Diagnosis of Malaria

Two and a half mL of venous blood was obtained from each patient. Malaria was diagnosed using blood film microscopy and confirmed with PCR. Both thick and thin blood films were used, fields were read at least twice, and the procedure was followed according to quality control guidelines of WHO. PCR was done for both* P. falciparum* and* P. vivax*. The PCR was performed at the Institute of Endemic Diseases, University of Khartoum, with quality control in place (both positive and negative control were used). Parasite genomic DNA was extracted from whole blood samples using Chelex method. A fragment of the plasmodial 18S rRNA gene was amplified by PCR and species identification was performed with species-specific oligoprobes using the following primers: for* P. falciparum*, rPLU5: 5′ CTTGTTGTTGCCTTAAACTTC-3′, rPLU6: 5′-TTAAAATTGTTGCATTAAAACG-3′; for* P. vivax*, rVIV1: 5′-CGCTTCTAGCTTAACCACATAACTGATAC-3′, rVIV2: 5′-ACTTCCAAGCCGAAG CAAAGA AAG TCC TTA-3′, as described previously [7].

### 2.4. Statistical Analysis

Data were analyzed using SPSS (statistical package for the social sciences) version twentieth software.

## 3. Results

In our study, both males and females are affected by malaria; however more females were represented in Aljabalain hospital (64.4%) and more males were represented from Aljabalain military hospital (64%). The average haemoglobin level in all patients from the two study sites was 11.9 g/dL. The prevalence of anaemia among malaria patients was high, more than two-thirds in both study sites. The majority of malaria cases (more than 90%) had low parasite level (≤500 parasite/*μ*L of blood), Table 1.

**Figure 2:**
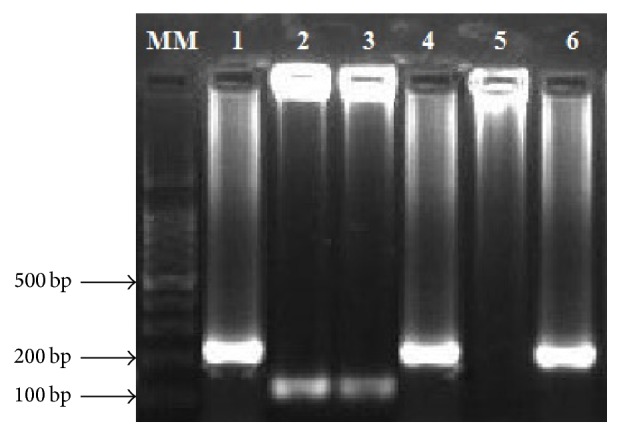
Detection of* Plasmodium* 18S rRNA gene using nested PCR from Sudanese malaria patients; MM: 100 bp ladder (iNtRON Biotechnology, South Korea), 1, 4, 6:* P*. falciparum, 2, 3:* P*.* vivax*, and 5: negative control.

In both study sites, the proportion of* P. vivax* infections among suspected malaria cases was high. Microscopy results showed that 30 (15%) and 50 (25%) of malaria infections in Aljabalain hospital and Aljabalain military hospital were caused by* P. vivax*. The results were even higher with PCR (Figure 2); 66 (33%) and 80 (40%) of samples from Aljabalain hospital and Aljabalain military hospital were positive for* P. vivax,* respectively. Mixed infections (*P. falciparum* +* P. vivax*) were detected in 2.25% of samples in average from both study areas, Table 2.

## 4. Discussion

This study was carried out in an area characterized by seasonal and unstable malaria transmission. The most remarkable result in this study was the unexpected high proportion (about 40% by PCR) of* P. vivax* infections among suspected malaria cases, eight times more than that previously reported in Sudan [4]. This change in pattern is most likely due to the recent varied composition of the community resulting from several migrations of people from several Asian and African countries to work at petroleum and new sugar companies in White Nile area especially from Ethiopia where high prevalence of* P. vivax* infection (31%) among malaria cases was found [6].

Another suggested explanation for the emergence of* P. vivax* is parasite's development of alternative mechanisms to invade human erythrocytes other than the Duffy antigen. This is a plausible explanation since* P. vivax* infection of Duffy negative genotype was reported previously in many African countries [8–16]. This is the first study of its kind to document the significant rise in malaria* P. vivax* transmission in Sudan. And it has important health policy implications since* P. vivax* infection requires eradication of liver stages with primaquine due to presence of dormant hypnozoites within hepatocytes [17, 18]. Recent data showed that* P. vivax* infection is becoming more severe especially in children, and further studies are required to understand the exact causes of this pattern [19].

The sampled population (four hundred cases) of this study was selected based on their highly suspected symptoms of malaria and positive blood film microscopy done in hospitals' laboratories. Blood films were later proved only 50% positive in more quality insured settings. This obvious result of malaria overdiagnosis by a factor of two is probably due to lack of training in general hospitals, low quality control microscopy, and high work load. The same result was previously found in Tanzania where malaria was overdiagnosed by a factor of five [20, 21].

PCR diagnosis for malaria is accurate especially for differentiating between plasmodia species, but it is expensive and needs well-trained personnel. In this study quality control microscopy allowed for 20% of* Plasmodium vivax* diagnosis, while by PCR it was 38.7%. This may be due to difficulty in differentiation between species based on a single ring form especially for untrained personnel. In this study more than 90% of slides had low parasite density (≤500 parasite/*μ*L); this also makes differentiation between plasmodia species very difficult.

## 5. Conclusion

Our study confirmed the observed high percentage of* P. vivax* infections in White Nile area, central Sudan. This result has important implications for the malaria control and necessitates modification of current guidelines for the treatment of malaria in Sudan.

## Figures and Tables

**Table 1:** The mean age, gender and haemoglobin level in Aljabalain hospital and military hospital.

	Aljabalain hospital	Military hospital
Number of patients	200	200
Mean age ± SD, range (years)	30 ± 15 (2–80)	31 ± 13 (1–70)
Less than 15 (%)	19 (9.5%)	17 (8.5%)
15–30 (%)	93 (46.5%)	92 (46.3%)
More than 30 (%)	88 (44%)	90 (45.2%)
Gender		
Males (%)	70 (34.7%)	128 (64%)
Females (%)	130 (64.4%)	72 (36%)
Mean Hb level ± SD, range (g/dL)	11.8 ± 2.2 (4–18)	12 ± 2.1 (5–19)
Anaemia^*∗*^ (%)	135 (67.5%)	146 (73%)
Parasitaemia^*∗∗*^		
Low (%)	180 (90%)	185 (92.5%)
High (%)	20 (10%)	15 (7.5%)

^*∗*^Anaemia was defined as Hb level less than 13.5 g/dL for males and 12.5 g/dL for females.

^*∗∗*^Low parasitaemia was defined as number of asexual parasites ≤500/*µ*L of blood. High parasitaemia: >500 asexual parasites/*µ*L of blood.

**Table 2:** Positive results of blood films and PCR for *P*. *falciparum* and *P*. *vivax* in Aljabalain hospital and military hospital.

	Aljabalain hospital (200)		Military hospital (200)	
	Microscopy	PCR	Microscopy	PCR
	*N* (%)	*N* (%)	*N* (%)	*N* (%)
*P*. *falciparum*	107 (53.5%)	115 (57.5%)	93 (46.5%)	112 (56%)
*P*. *vivax*	30 (15%)	66 (33%)	50 (25%)	80 (40%)
Mixed (*P*. *falciparum* + *P*. *vivax*)	0 (0%)	5 (2.5%)	0 (0%)	4 (2%)
Negative	63 (31.5%)	14 (7%)	57 (28.5%)	4 (2%)

## Abbreviations

BFFM: Blood film for malaria
PCR: Polymerase chain reaction
Pf: * Plasmodium falciparum*
Pv: * Plasmodium vivax*.

## References

[1] Centers for Disease Control and Prevention Malaria Worldwide. http://www.cdc.gov/malaria/malaria_worldwide/index.html.

[2] WHO Malaria. http://www.who.int/mediacentre/factsheets/fs094/en/.

[3] Baird J. K. (2013). Evidence and implications of mortality associated with acute plasmodium vivax malaria. *Clinical Microbiology Reviews*.

[4] Mendis K., Sina B. J., Marchesini P., Carter R. (2001). The neglected burden of *Plasmodium vivax* malaria. *The American Journal of Tropical Medicine and Hygiene*.

[5] Golassa L., Baliraine F. N., Enweji N., Erko B., Swedberg G., Aseffa A. (2015). Microscopic and molecular evidence of the presence of asymptomatic *Plasmodium falciparum* and *Plasmodium vivax* infections in an area with low, seasonal and unstable malaria transmission in Ethiopia. *BMC Infectious Diseases*.

[6] Lo E., Yewhalaw D., Zhong D. (2015). Molecular epidemiology of Plasmodium vivax and Plasmodium falciparum malaria among duffy-positive and duffy-negative populations in Ethiopia. *Malaria Journal*.

[7] Snounou G., Singh B. (2002). Nested PCR analysis of Plasmodium parasites. *Methods in molecular medicine*.

[8] Sondén K., Castro E., Trönnberg L., Stenström C., Tegnell A., Färnert A. (2014). High incidence of Plasmodium vivax malaria in newly arrived Eritrean refugees in Sweden since may 2014. *Eurosurveillance*.

[9] Mathews H. M., Armstrong J. C. (1981). Duffy blood types and vivax malaria in Ethiopia. *American Journal of Tropical Medicine and Hygiene*.

[10] Ménard D., Barnadas C., Bouchier C. (2010). Plasmodium vivax clinical malaria is commonly observed in Duffy-negative Malagasy people. *Proceedings of the National Academy of Sciences of the United States of America*.

[11] Mendes C., Dias F., Figueiredo J. (2011). Duffy negative antigen is no longer a barrier to Plasmodium vivax—molecular evidences from the African West Coast (Angola and Equatorial Guinea). *PLoS Neglected Tropical Diseases*.

[12] Mercereau-Puijalon O., Ménard D. (2010). Plasmodium vivax and the Duffy antigen: a paradigm revisited. *Transfusion Clinique et Biologique*.

[13] Ngassa Mbenda H. G., Das A. (2014). Molecular evidence of *Plasmodium viva*x mono and mixed malaria parasite infections in Duffy-negative native Cameroonians. *PloS one*.

[14] Ryan J. R., Stoute J. A., Amon J. (2006). Evidence for transmission of Plasmodium vivax among a Duffy antigen negative population in Western Kenya. *American Journal of Tropical Medicine and Hygiene*.

[15] Woldearegai T. G., Kremsner P. G., Kun J. R. F. J., Mordmüller B. (2013). Plasmodium vivax malaria in duffy-negative individuals from Ethiopia. *Transactions of the Royal Society of Tropical Medicine and Hygiene*.

[16] Wurtz N., Mint Lekweiry K., Bogreau H. (2011). Vivax malaria in Mauritania includes infection of a Duffy-negative individual. *Malaria Journal*.

[17] Fernando D., Rodrigo C., Rajapakse S. (2011). Primaquine in vivax malaria: an update and review on management issues. *Malaria Journal*.

[18] Hulden L., Hulden L. (2011). Activation of the hypnozoite: a part of *Plasmodium vivax* life cycle and survival. *Malaria Journal*.

[19] Mahgoub H., Gasim G. I., Musa I. R., Adam I. (2012). Severe *Plasmodium vivax* malaria among Sudanese children at New Halfa Hospital, Eastern Sudan. *Parasites & Vectors*.

[20] Harchut K., Standley C., Dobson A. (2013). Over-diagnosis of malaria by microscopy in the Kilombero Valley, Southern Tanzania: an evaluation of the utility and cost-effectiveness of rapid diagnostic tests. *Malaria Journal*.

[21] Reyburn H., Mbatia R., Drakeley C. (2004). Overdiagnosis of malaria in patients with severe febrile illness in Tanzania: A Prospective Study. *British Medical Journal*.

